# Tooth Eruption and Microbial Correlation in Pediatric Appendicitis: An Exploratory Case–Control Study

**DOI:** 10.3390/jcm14207372

**Published:** 2025-10-18

**Authors:** Wieland Elger, Carlotta Blod, Sara Schülin, Christian Hirsch, Martin Lacher, Steffi Mayer

**Affiliations:** 1 Department of Pediatric Dentistry and Primary Prevention, University Leipzig, 04103 Leipzig, Germany; 2 Department of Pediatric Surgery, University Hospital Leipzig, 04103 Leipzig, Germany

**Keywords:** pediatric appendicitis, dental characteristics, dental development, oral sampling sites, oral microbiota

## Abstract

**Background/Objectives**: The oral cavity has been discussed as a possible reservoir for pathogens involved in pediatric appendicitis. In a previous study, microbial similarities between oral and appendix samples were observed, but clinical dental findings showed no significant associations. The present study aimed to (1) perform a more detailed analysis of dental variables—such as eruption status, caries indices, oral hygiene behavior, and the Periodontal Screening Index (PSI)—to identify potential overlooked associations with appendicitis, and (2) compare two oral sampling sites (buccal mucosa and dental sulcus) in terms of their correlation with appendix microbiota. **Methods**: This secondary analysis used previously collected clinical and microbiological data from 36 children who had undergone appendectomy or elective surgery. Quantitative PCR (qPCR) was used to assess bacterial mRNA levels in appendix, buccal mucosa, and sulcus samples. Dental variables were derived from standardized clinical examinations performed at the time of surgery. **Results**: No significant group differences were observed in caries indices, PSI, or oral hygiene behavior. However, patients with appendicitis had a significantly higher number of erupting teeth compared to controls (3.82 vs. 1.68; *p* = 0.021). Bacterial mRNA levels did not differ notably between oral sites, and neither showed stronger correspondence with appendix samples; notably, sulcus swabs were not obtained from erupting teeth, limiting the ability to test a site-specific eruption mechanism. **Conclusions**: Tooth eruption may be associated with appendicitis, although this remains speculative and requires confirmation in larger studies.

## 1. Introduction

This study is a single-center, exploratory case–control investigation using data collected between January and June 2015. The sample is relatively small (n = 36) and focuses on a narrow phenotype (children undergoing appendectomy for acute phlegmonous appendicitis), which limits generalizability. The relationship between oral health and general health is well documented [1]. Recent systematic reviews and large-scale studies highlight that oral conditions, including periodontitis and dental infections, can influence systemic diseases such as cardiovascular disorders, chronic kidney disease, and inflammatory bowel disease [2,3,4,5]. Accumulating evidence highlights the importance of oral–systemic interactions, indicating that changes in the oral microbiota can influence systemic inflammation and may be linked to conditions beyond the oral cavity [6,7,8]. Based on these observations, we aimed to investigate whether specific dental parameters and oral microbiota are associated with pediatric appendicitis.

In particular, a possible link with oral microorganisms has been suggested in relation to acute appendicitis in childhood, although the specific types of bacteria involved have not yet been clearly identified [9,10,11]. Our research group has shown that certain oral bacteria, such as *Peptostreptococcus stomatis*, can be detected in both the oral cavity and the appendix of patients with appendicitis. In addition, their levels correlated with inflammatory parameters such as leukocyte count and C-reactive protein [1]. Other experimental studies have also shown that selected oral species can survive passage through the stomach, suggesting possible migration from the oral cavity into the gastrointestinal tract [11]. There is also evidence that oral bacteria can persist in the gastrointestinal tract and promote microbial dysbiosis and immunological dysregulation [10,12]. These findings support the hypothesis that the oral cavity may be a reservoir for pathogenic bacteria involved in the development of appendicitis. In our previous study from 2015, the results indicated viable migration from the oral cavity through the stomach to the appendix. Therefore, the oral cavity could be a relevant reservoir for acute appendicitis. The dental characteristics of the appendicitis patients, which included factors relating to dental check-ups, tooth brushing behavior and the frequency of the presence of decayed and restored teeth, as well as the Periodontal Screening Index (PSI), did not differ significantly from those of the control group [1]. However, it seems appropriate to analyze these data in more detail. An extended analysis with other previously collected dental factors might also help to identify differences that have been missed so far.

Another challenge in researching this relationship is determining the most appropriate oral sampling site for microbiological analysis. In our study, swabs were taken from the buccal mucosa and the dental sulcus. Only data from the sulcus were included in the data analysis. However, it remains unclear which of the two sampling sites—the sulcus or the buccal mucosa—has a better correlation with the microbiota of the appendix. Different areas of the oral cavity harbor different microbial communities. For example, the dental sulcus provides an anaerobic environment that is influenced by the gingival sulcular fluid and thus characterizes the bacterial composition [13,14,15,16]. In contrast, a buccal swab, which is less invasive, may provide a different microbial profile [15,16,17].

The present study has two main aims: first, to perform a more detailed analysis of the dental findings in order to identify possible previously overlooked associations with appendicitis and, second, to investigate which of the two oral sampling sites—the dental sulcus or the buccal mucosa—shows a stronger correlation with the appendix microbiota.

## 2. Materials and Methods

### 2.1. Study Design and Participants

This single-center, exploratory case–control study was conducted using prospectively collected data from January to June 2015 [1]. The appendicitis group included children and adolescents undergoing laparoscopic appendectomy for acute phlegmonous appendicitis, whereas the control group comprised children admitted for elective surgical procedures. The age range of included patients was 3 to 17 years. Patients undergoing interval appendectomy, complicated appendicitis (perforation, abscess), or with a history of chronic abdominal pain, gastrointestinal disorders, or prior appendectomy were excluded. Preoperative antibiotics were administered only for suspected perforated appendicitis. All patients were offered a standardized dental examination by a pediatric dentist. A total of 36 patients were included; however, complete triplicate samples (buccal mucosa, sulcus, appendix) were not available for all participants, which explains the ‘not calculable’ correlations in some analyses. Written informed consent was obtained from the parents of all participants.

### 2.2. Dental Examinations

In addition to various medical evaluations, the oral cavity was inspected. The dental assessments in the pediatric surgical ward used standard instruments such as a mirror, a probe, and a WHO probe. Dental cotton rolls were applied to dry the teeth during this process. The results were recorded on a dental chart. In addition to recording the number of teeth and caries status using the DMF/T index (decayed, missing, filled teeth) for permanent and primary teeth, the eruption status of the teeth was assessed. According to the criteria of Carvalho et al. [18], teeth were classified as erupting if they were not fully occluded or if the vestibular crown sur-face was still covered by gingiva. To assess the periodontal health of the participants, we determined the Periodontal Screening Index (PSI), which ranges from 0 to 4, by probing the depth of the gingival pocket, assessing the bleeding tendency, and checking the presence of calculus. A PSI score of ≥3 indicates the presence of periodontitis, which requires comprehensive periodontal treatment. Erupting teeth were not included in the PSI assessment. Furthermore, a questionnaire was completed about the frequency of dental visits (“no” or “yes, regularly”) and tooth brushing habits (“never,” “once,” “twice,” “three times a day”).

### 2.3. Bacterial Sample Collection and Processing

Samples were taken preoperatively from the sulcus as described by Blod et al. [1]. Sterile paper tips (ISO 50, Roeko-GmbH, Langenau, Germany) were used and inserted into four different tooth sulci for 30 s. The buccal mucosa was swabbed with an OmniSwab (QIAGEN GmbH, Hilden, Germany). All samples were immediately transferred to individual sterile, media-free Eppendorf tubes and stored at −80 °C until further analysis. During laparoscopic appendectomy, the appendix was opened under sterile conditions and swabs were taken from the intraluminal side and also cryopreserved at −80 °C [1]. After thawing, bacterial DNA was extracted by enzymatic digestion, mechanical disruption and purification using the QIAamp DNA Mini/Micro Kit (QIAGEN, Hilden, Germany). DNA quality and quantity were assessed using a spectrophotometer. For microbiome analysis, the V1-V3 regions of the 16S rDNA genes were amplified and sequenced using the Illumina MiSeq platform, followed by sequence processing and taxonomic assignment using the QIIME software package (Version 1.8.0). Based on prior evidence of their presence in the oral microbiome and our preliminary findings, four bacterial species (*Fusobacterium nucleatum*, *Eikenella corrodens*, *Peptostreptococcus stomatis*, and *Fusobacterium periodonticum*) were selected for further analysis by reverse transcription quantitative PCR (RT-qPCR) to quantify bacterial mRNA expression levels. Primers and probes were designed as previously described [1]. RNA was extracted from the same swabs using the QIAamp RNA Mini Kit, and RNA quality and concentration were assessed with a NanoDrop spectrophotometer. cDNA synthesis was performed using random hexamers and reverse transcriptase. Quantitative PCR results were normalized to total RNA input, and mRNA expression levels—rather than DNA copy number—were used for correlation analyses with appendix samples [1].

### 2.4. Statistical Analyses

The statistical analysis was conducted using Microsoft Excel 2016 and IBM SPSS Statistics Version 29. Following the assessment of continuous data for normal distribution, parametric distribution was analyzed using the two-sided t-test (for age at surgery, body mass index (BMI)), and nonparametric distribution (PSI) was examined by the Mann–Whitney U test. Statistical significance was attributed to *p*-values < 0.05. Logistic regression was used to analyze the association between number of erupting teeth and appendicitis, adjusting for age and sex as potential confounders. Potential confounders were considered, collinearity was assessed using Variance Inflation Factors (VIF), and model stability was evaluated through sensitivity analyses.

To assess associations between bacterial mRNA levels at different sampling sites and the appendix, Spearman’s rank correlation coefficients (r_s_) were calculated for paired samples (buccal mucosa and sulcus vs. appendix) for each bacterial species. Spearman’s r_s_ ranks the values and quantifies the degree of monotonic association between paired observations. *p*-values were calculated to determine statistical significance (*p* < 0.05). In addition, the size of r_s_ was interpreted as an effect size, with values categorized as small (r_s_ < 0.3), medium (r_s_ = 0.3–0.5) and large (r_s_ > 0.5). These measures provide information on the statistical and practical significance of the results. In addition, descriptive statistics for oral bacterial mRNA levels were reported for healthy control patients. In addition, descriptive statistics (median and interquartile range, IQR) for oral bacterial mRNA levels are reported, allowing comparison between buccal mucosa and sulcus.

## 3. Results

The present study comprised a total of 36 children, of whom 21 (58.3%) were male. The mean age of the participants was 10.48 ± 3.68 years. The majority of children (n = 25) reported brushing their teeth at least twice daily, while 11 brushed less frequently, and only one child did not undergo regular dental check-ups, as summarized in Table 1.

As demonstrated in Table 2, no statistically significant differences were observed between the appendicitis and control groups with regard to age (*p* = 0.440), sex (*p* = 0.158), tooth brushing frequency (*p* = 0.900), or total number of teeth (both groups: ~24 teeth). The appendicitis group exhibited a marginally higher number of deciduous teeth (7.76 ± 7.47 vs. 5.42 ± 7.86), though considerable variability was observed in both groups. The periodontal screening index (PSI) and DMF/T index are reported as median (interquartile range, IQR). No significant differences were observed between groups.

The only significant difference observed between the groups pertained to the number of teeth in the eruptive stage (*p* = 0.021). Patients with appendicitis exhibited a significantly higher number of erupting teeth in comparison to the control group (3.82 vs. 1.68). This association remained significant in the logistic regression model (*p* = 0.025, Table 3), with an odds ratio of 1.42 (95% CI: 1.04–1.94), indicating that a higher number of erupting teeth was associated with an increased likelihood of appendicitis. Table 4 presents bacterial mRNA expression levels (RT-qPCR) in appendicitis patients for three sampling sites: buccal mucosa, sulcus, and appendix. The median and interquartile range (IQR) are reported for each sample type. Additionally, Spearman correlation coefficients (r) and corresponding *p*-values are provided to assess the relationship between oral sampling sites and appendix values. No statistically significant correlations were observed between oral and appendix samples. Paired counts were limited, with n = 7–8 for *E. corrodens* and *P. stomatis* correlations, and only 2–3 for *F. nucleatum* and *F. periodonticum*. Given the sparse data, these analyses should be regarded as purely exploratory.

## 4. Discussion

This exploratory case–control study did not identify a significant correlation between classic dental findings and appendicitis. However, a statistically significant association was found with the number of erupting teeth—a standard parameter in dental examinations used to monitor developmental changes. Epidemiological data show that the incidence of appendicitis tends to increase from around age six—coinciding with the eruption of the first permanent molars—and peaks during the mixed dentition phase [19,20,21,22]. Whether this reflects a mere correlation or an underlying causal mechanism remains unclear. In particular, tooth eruption represents a developmental phase accompanied by local inflammatory processes and microbial shifts that may contribute to systemic susceptibility. Given that the incidence of pediatric appendicitis peaks during the mixed dentition phase, the interplay between erupting teeth, associated microbial changes, and immune activation deserves closer investigation [16,23,24,25].

It is important to note that, due to the case–control design, causality cannot be established. Observed associations should therefore be interpreted as exploratory, and the influence of unidentified or uncontrolled confounders—such as dietary habits, oral hygiene practices, prior antibiotic exposure, or systemic inflammatory status—cannot be excluded. The limited sample size and the small number of complete triplicate samples (buccal mucosa, sulcus, appendix) constrain the interpretability of our microbial findings and may obscure subtle correlations. Paired oral–appendix samples were very few, ranging from only 2–8 per bacterial species, so Spearman correlations cannot provide robust evidence of microbial overlap. Recruitment was inherently difficult due to the acute nature of appendicitis, which necessitates prompt surgical intervention and reduces the available time for study inclusion [26,27]. Multiple comparisons were made across species and oral sites; however, since no associations reached statistical significance, formal false-discovery rate correction was not applied. No prespecified primary organism was tested, and the results should therefore be regarded as exploratory and hypothesis-generating, rather than confirmatory. Thus, these factors should be considered when interpreting our findings.

Regarding microbial findings, no significant differences were detected between the sampling sites, raising questions about the suitability of different oral locations for reflecting potential microbial transmission. The gingival sulcus, influenced by gingival fluid, may offer a distinct microbial environment compared to the buccal mucosa, which contains fewer bacteria but more epithelial cells and saliva [15]. However, our findings contrast with studies reporting that the gingival sulcus harbors a more diverse microbiota than the oral mucosa [13,16].

Notably, our sulcus samples were not taken from the erupting teeth themselves, leaving the microbial environment at those sites unassessed. Eruption alters the oral ecosystem by affecting biofilm structure, nutrient availability, and oxygen levels, leading to shifts in the oral microbiota [28]. Previous studies have shown increased microbial diversity and plaque accumulation during the mixed dentition phase [16,28,29,30]. Reduced parental supervision during this period may further contribute to a higher bacterial load [31]. However, these microbial shifts likely influence the entire oral cavity rather than being restricted to specific sampling sites, which could explain the absence of clear differences in our data.

Interestingly, despite the observed association between tooth eruption and appendicitis, we found no strong correlation between the oral and appendix microbiota. We also clarify that the correlations with appendix microbiota were performed using bacterial mRNA levels rather than DNA copy number, which provides a measure of bacterial activity rather than mere presence. Notably, sulcus samples were not collected from erupting teeth, which limits the ability to test a site-specific microbial mechanism. This discrepancy may suggest that systemic factors rather than direct bacterial transmission play a role. Tooth eruption is known to trigger inflammatory cytokines such as IL-1β, IL-6, and TNF-α, which are involved in immune regulation [32,33]. Future studies should explore whether systemic inflammatory markers related to tooth development contribute to appendicitis susceptibility.

Our original hypothesis was not explicitly focused on erupting teeth but rather on general associations. The observed relationship emerged unexpectedly through standardized recording of eruption status. Larger cohorts and longitudinal studies are needed to investigate whether these findings hold clinical relevance. Additionally, further research should examine microbial shifts before and during the mixed dentition phase to clarify potential pathways linking oral and systemic health.

In summary, the study is limited by its small sample size, the exploratory design, and the potential impact of unmeasured confounders. These factors should be considered when interpreting the findings, and further research with larger, longitudinal cohorts is warranted to validate and clarify the observed associations.

## 5. Conclusions

Under the tested conditions, no significant correlations were observed between oral and appendix samples, and classic dental findings did not differ between groups. The observed association with erupting teeth suggests a potential developmental or systemic signal, but the small sample size and limited pairing restrict inference. Our results should therefore be regarded as hypothesis-generating, warranting confirmation in larger and longitudinal studies to further investigate possible biological mechanisms linking dentition stage and appendicitis.

## Figures and Tables

**Table 1:** Demographics of study population.

	N
Total	36
Mean age (SD ^1^)	10.48 (±3.68)
Sex (%)	
Male	21 (58.3)
Female	15 (41.7)
Tooth brushing frequency (%)	
Never	2 (5.6)
Once daily	9 (25.0)
Twice daily	22 (61.1)
Three times daily	3 (8.3)
Regular dental check-ups (%)	
No	1 (2.8)
Yes	35 (97.2)
Teeth erupting (%)	
No	16 (44.4)
Yes	20 (55.6)
Mean PSI ^2^ (SD)	1.14 (±0.87)
Mean dmf/t ^3^ + DMF/T ^4^ (SD)	1.25 (±2.74)

^1^ SD, Standard deviation. ^2^ PSI, Periodontal Screening Index. ^3^ dmf/t, decayed/missing/filled primary teeth. ^4^ DMF/T, Decayed/Missing/Filled permanent Teeth.

**Table 2:** Distribution of Recorded Variables Between Study and Control Groups.

	Appendicitis Patients	Healthy Controls	*p*-Value
Age (year)	10.94 (±4.10)	9.97 (±3.19)	0.440 ^†^
Sex (m:f)	12 (70.6%):5 (29.4%)	9 (47.4%):10 (52.6%)	0.158 ^#^
Tooth brushing	1.71 (±0.85)	1.74 (±0.56)	0.900 ^†^
Regular dental check-ups	17 (100%)	18 (94.74%)	0.337 ^#^
Eruption (Number of teeth)	3.82 (±3.50)	1.68 (±2.54)	0.021 ^†^
Number of teeth	24.18 (±2.35)	24.53 (±4.43)	0.383 ^†^
Number of primary teeth	7.76 (±7.47)	5.42 (±7.86)	0.367 ^†^
PSI ^1^ (median, IQR)	1.00 (1.92)	1.33 (1.50)	0.203 ^†^
BMI ^2^	18.68 (±4.32)	20.07 (±5.52)	0.205 ^†^
dmf/t ^3^ + DMF/T ^4^ (median, IQR)	2.00 (4)	0.50 (2)	0.590 ^††^

Values are presented as mean (±standard deviation) for normally distributed continuous variables or median (interquartile range, IQR) for ordinal/zero-inflated variables. ^1^ PSI, Periodontal Screening Index; ^2^ BMI, Body Mass Index; ^3^ dmf/t, decayed/missing/filled primary teeth; ^4^ DMF/T, Decayed/Missing/Filled permanent teeth; ^†^ T-Test for normally distributed continuous variables; ^#^ Chi-square-Test for categorical variables; ^††^ Mann–Whitney U Test for non-parametric/ordinal variables.

**Table 3:** Multivariable logistic regression analysis of factors associated with acute pediatric appendicitis.

	OR ^2^	95% CI ^3^	*p*-Value
Age (year)	0.93	0.75–1.14	0.480
Sex (m:f)	1.69	0.34–8.31	0.519
Eruption (Number of teeth)	1.42	1.04–1.94	0.025
PSI ^1^ (mean)	0.37	0.12–1.15	0.085

^1^ PSI, Periodontal Screening Index; ^2^ OR, Odds Ratio; ^3^ CI, Confidence Interval.

**Table 4:** Spearman correlation coefficients between bacterial mRNA levels in oral samples (buccal mucosa, sulcus) and the appendix.

Bacterium		Median (IQR ^1^)		r_s_ ^2^	*p*-Value	n Paired	r_s_ ^2^	*p*-Value	n Paired
	Buccal Mucosa	Sulcus	Appendix	Buccal vs. Appendix			Sulcus vs. Appendix		
*Eikenella corrodens*	9.34 (0.69)	7.51 (1.32)	3.39 (4.46)	0.143	0.760	7	−0.643	0.119	7
*Peptostreptococcus stomatis*	3.51 (2.80)	4.60 (1.04)	5.52 (5.26)	0.143	0.736	8	−0.107	0.819	7
*Fusobacterium* *nucleatum*	1.86 (0.02)	3.09 (3.51)	2.12 (0.95)	nc ^3^	-	2	nc ^3^	-	2
*Fusobacterium* *periodonticum*	1.15 (1.15)	1.46 (1.49)	1.03 (2.07)	nc ^3^	-	2	nc ^3^	-	3

^1^ IQR, interquartile range; ^2^ r_s_, Spearman’s rank correlation coefficients; ^3^ nc, Not calculable (Due to insufficient sample size for paired analysis).

## Data Availability

The data presented in this study are available on request from the corresponding author.

## Abbreviations

The following abbreviations are used in this manuscript:

PSI	Periodontal Screening Index
DMF/T	Decayed, Missing, Filled Teeth
DNA	Deoxyribonucleic Acid
rDNA	Ribosomal Deoxyribonucleic Acid
QIIME	Quantitative Insights Into Microbial Ecology
RT-qPCR	Reverse Transcription quantitative Polymerase Chain Reaction
BMI	Body Mass Index
mRNA	Messenger Ribonucleic Acid
r_s_	Spearman’s rank correlation coefficient
SD	Standard Deviation
IQR	Interquartile Range
CI	Confidence Interval
IL-1β	Interleukin 1 beta
IL-6	Interleukin 6
TNF-α	Tumor Necrosis Factor alpha
